# Physicochemical and Antioxidant Properties of Wheat Bread Enriched with Hazelnuts and Walnuts

**DOI:** 10.3390/foods9081081

**Published:** 2020-08-08

**Authors:** Karolina Pycia, Eva Ivanišová

**Affiliations:** 1 Department Food Technology and Human Nutrition, Institute of Food Technology, College of Natural Science, University of Rzeszow, Zelwerowicza 4 St., 35-601 Rzeszow, Poland; 2 Department of Technology and Quality of Plant Products, Faculty of Biotechnology and Food Sciences, Slovak University of Agriculture in Nitra, Tr. A. Hlinku 2, SK-949 76 Nitra, Slovakia; eva.ivanisova@uniag.sk

**Keywords:** bread, nuts, enrichment

## Abstract

The aim of this study was to evaluate the effect of wheat bread enrichment using hazelnuts and walnuts (1%, 3%, 6%, 9%) on the nutritional value and selected physicochemical and antioxidant properties. The dough and bread yield, volume, specific volume and porosity were also determined. The crumb texture was analyzed by texture profile analysis (TPA) test using a texture meter. The color of the crumb was assessed in the CIE L*a*b* color space. Antioxidant properties were determined by the ABTS^+^ radical method. The contents of phenolic acids, flavonoids and total polyphenols were also determined. The test demonstrated that the enrichment of bread with nuts increased the level of minerals, protein, fiber and fat. Breads containing walnuts were characterized by the highest content of these nutrients. The bread with a 9% walnut content by the smallest volume (380 cm^3^) had lowest value of L*. The crumb of the enriched breads was characterized by greater hardness, gumminess and chewiness, the values of these parameters generally increasing in parallel to the nut content. Breads enriched with walnuts were characterized by a higher average total content of polyphenols (35.77 mg gallic acid equivalent (GAE)/100 g dry mass (DM)) compared to the breads enriched with walnuts (25.35 mg GAE/100 g DM).

## 1. Introduction

The consumers’ expectations concerning food is that, in addition to its primary role of satisfying hunger, it should feature high nutritional value, deliciousness and ease of access. An example of a cereal product that meets these expectations is bread. The availability of bread, along with its volume of consumption and ease of production, means that it is a product suitable for enrichment [1]. In breads made from ‘light’ wheat flour, the main nutrients and dietary fiber content is significantly lower than in breads made from whole grain flour. The enrichment of bread with plant materials with high health-promoting potential could increase the supply of bioactive ingredients in the diet. Nuts are one plant material that could fulfil this role. Due to their chemical composition and related nutritional value, nuts are considered to be functional food. Nut seeds form a rich, natural source of fat, including unsaturated fatty acids, protein, carbohydrates, minerals (potassium, magnesium, selenium), fat-soluble vitamins (especially E, A) and B vitamins (B1, B2). Moreover, they contain bioactive ingredients such as carotenoids, phytosterols, polyphenols and other substances with a strong antioxidant potential. Therefore, the daily consumption of nuts contributes to reducing the risk of diet-related diseases, such as obesity, diabetes, cardiovascular diseases and cancer [2,3]. Of all the edible nuts, hazelnuts and walnuts have the highest nutritional value. Walnuts are the richest source of polyunsaturated fatty acids and have the most favorable ratio of n-6 and n-3 fatty acids, which should be 4:1. Hazelnuts and almonds are an excellent source of vitamin E [4]. According to Kornsteiner et al. [5], a portion of hazelnuts (42 g) provides more than 100% of the recommended daily intake of vitamin E, so that the body is protected against free radicals, thus preventing early ageing, atherosclerosis and cancer. The literature provides information on the dough properties and quality of bread enriched with oilseed [6], manioc and mushroom flour [7], black oat flour [8], chia seeds [9], grape pomace [10] or walnut flour [1]. In all cases, the quoted authors observed changes in the rheological properties of the dough, the nutritional value of the bread, the color and texture of the crumb, and the antioxidant properties and sensory qualities resulting from the addition of plant components. There are, however, no data available on the changes in the quality of wheat bread due to enrichment with ground walnuts and hazelnuts in different quantities. This type of bread can be an example of a functional product with strong antioxidant properties, which is not guaranteed by the addition of other plant materials.

The aim of the study was to evaluate the effect of wheat bread enrichment with walnuts and hazelnuts on their nutritional value, selected physicochemical parameters, texture profile and antioxidant properties.

## 2. Materials and Methods

### 2.1. Materials

The research material included wheat breads enriched with ground dry hazelnut and walnut seeds. The fat content of hazelnuts and walnuts was 57% and 65%, respectively. The hazelnut and walnut seeds were ground in a Cemotec mill (Foss, Helsingborg, Sweden). Wheat flour (wet gluten yield: 27.0%, total protein content: 11.5%) type 550 with a water absorption of 61% and a dry matter content of 13.0% was used as the basis for the bread. The flour was obtained as a result of the laboratory milling in the Quadrumat Junior mill (Brabender, Duisburg, Germany) of wheat grain previously conditioned to a humidity of 15%. The analysis involved replacing some of the wheat flour by ground hazelnuts and walnuts, in the amounts 1%, 3%, 6% and 9% in relation to the flour by weight. The control sample was wheat bread made from wheat flour only (0% nut content). The tests were repeated three times. The bread was baked from a single-phase dough [11,12].

#### Laboratory Baking

The dough was prepared from wheat flour, baker’s yeast (Lallemand, Józefów, Poland) (3% by weight of flour used), sodium chloride (1% by weight of flour used), water, as well as ground walnut and hazelnut seeds, using a laboratory mixer (Mesko-AGD, Skarżysko-Kamienna, Poland). After mixing all the ingredients of the recipe, the dough was fermented for 60 min at a temperature of 30 °C, with a simultaneous puncture after 30 min. Afterwards, the billets of 250 ± 1 g weight were formed and placed in molds. The optimal time for the final dough expansion was 50 min. The bread was baked using an electric modular oven (Sveba Dahlen, Fristad, Sweden) at 230 °C, for 30 min [12,13].

### 2.2. Methods

#### 2.2.1. Laboratory Baking Parameters

Twenty-four hours after baking, the parameters for the laboratory produced bread products were determined, such as dough yield (%), bread yield (%), and total oven loss (%) [12,14]. Moreover, the volume of bread (cm^3^) was determined with the Sa-Way apparatus, using loose millet seeds [12,13]. The specific volume, i.e., the volume of bread per 1 g of flour used (cm^3^/g) [15] was also calculated and the porosity of bread was determined using the Dallman scale [16]. The determinations were made in two repetitions.

#### 2.2.2. Nutritional Assessment of Breads

The analyzed breads were subjected to nutritional assessment to determine the total mineral content (ash), protein, fiber and fat. The approximate composition of bread samples was determined following the official standard method (The Association of Official Analytical Chemists—AOAC) [17]. Protein was determined by the Kjeldahl method (AOAC 988.05) [17]. Oil and ash were estimated by the Soxhlet method (AOAC 963.15) [17] and drying methods (AOAC 942.05) [17], respectively. Crude fiber content was evaluated by the Ancom200 Fiber Analyzer (Ancom. Technology, Fairport, NY, USA) according to the producer—one gram (W_2_) of the sample was weighed in a special filter bag (W_1_; F57, Ancom, Fairport, NY, USA). The samples were defatted with petroleum ether, air-dried and placed into the analyzer; 2000 mL of 0.1 M sulphuric acid was added, and the samples were hydrolyzed at 100 °C for 45 min. Subsequently, the samples were washed with hot distilled water 3 times. Afterwards, 2000 mL of 0.1 M potassium hydroxide were added, and the samples were hydrolyzed at 100 °C for 45 min. The samples were then washed with hot distilled water 3 times. Water was gently pressed from the bags and the samples were soaked in acetone for 5 min, removed, air-dried and placed into the oven at 105 °C (WTB, Binder, Tuttlingen, Germany) for 2 h. After cooling to room temperature, the bags were re-weighted and ashed at 550 °C in pre-weighted crucibles for 2 h. The ashed crucibles were weighed to calculate the loss of weight of the organic matter (W_3_). Crude fiber content (%) was calculated using the following Equation (1):

(1) $$crude\;fiber\;content=\frac{W_{3}-\left(W_{1}\times \;C_{1}\right)}{W_{2}}\times 100$$

where:

C_1_>1—ash-corrected blank bag factor (running average of loss of weight on ignition of blank bag/original blank bag),
W_1_—bag tare weight (g),
W_2_—weight of sample (g),
W_3_—weight after ashing (g)

All markers were checked in three repetitions.

#### 2.2.3. Bread Crumb Color

Twenty-four hours after baking, the crumb of the wheat bread enriched to various extents with walnuts and hazelnuts was subjected to color analysis using a spectrophotometer (HunterLab, Reston, VA, USA). The measurements were carried out using the CIE L*a*b* system, in reflection: measuring geometry d/8°, illuminant D65, range 400–700 nm, detector tilted by 10°, and measuring slot 25 mm. Parameter L* should be in the range 0 to 100, where 0 indicates ‘zero’ luminance, i.e., black color, whilst 100 indicates the brightness of a spectral color, i.e., a monochromatic color. Parameters a and b determine the chromaticity of the color. Parameters a* and b* take values between −120 and 120, and indicate the transition from green (−a) to red (+a) and from blue (−b) to yellow (+b), respectively [12,18]. Parameter ΔE shows the calculated color difference [19]. All the measurements were made in five repetitions.

#### 2.2.4. Bread Crumb Texture Analysis

Twenty-four hours after baking the wheat breads, texture profile analysis (TPA) was carried out with the use of the EZ Test EZ-LX texture meter (Shimadzu, Kyoto, Japan) controlled by Trapezium X Texture Pl software. A crumb sample from the central part of the bread, cylindrical in shape (h = 27 mm, d = 32 mm, V = 22 cm^3^), was compressed twice along its axis with a 25 mm-diameter stainless steel disc at a speed of 50 mm·s^−1^ until 50% deformation was obtained. The following parameters of bread crumb texture were determined: hardness (N), cohesiveness (-), springiness (mm), gumminess (N) and chewiness (N). The measurements were taken in four repetitions.

#### 2.2.5. Analysis of Antioxidant Potential and Total Content of Phenolic Acids, Flavonoids and Polyphenols

The analyzed breads were dried with a freeze-drying unit (ALPHA 1-2 LD plus), and then the samples were ground. Extracts were made from the obtained lyophilisate using a 70% methanol solution. The extraction was carried out in an ultrasonic bath (Polsonic, Warszawa, Poland) for 30 min at 25 °C. The samples were centrifuged at 7000 rpm. Clear extracts were used for marking.

Antioxidant activity was determined in relation to the ABTS* cationic radical [20]. A total of 3 cm^3^ of ABTS* (2, 2’-azinobis (3-ethylbenzothiazoline-6-sulfonic acid)) radical was added to 0.03 cm^3^ of the extract. The value of the color absorbance of the solution was measured after 6 min of reaction using a Hitachi UV–VIS spectrophotometer type U-2900 (Hitachi, Tokyo, Japan) at wavelength λ = 734 nm. Antioxidant activity values were expressed in µmol TE/100 g dry mass (DM) (TE—Trolox Equivalent—α-tocopherol analogue).

Total phenolic acids content (TPAC) was determined using the method of Farmakopea Polska [21]. A 0.5 cm^3^ of extract was mixed with 0.5 cm^3^ of 0.5 M hydrochloric acid, 0.5 cm^3^ Arnova reagent (10% NaNO_2_ + 10% Na_2_MoO_4_), 0.5 cm^3^ of 1 M sodium hydroxide (*w*/*v*), and 0.5 cm^3^ of water. Absorbance at 490 nm was spectrophotometrically measured. A calibration curve was built using caffeic acid (R^2^ = 0.999). Results were expressed in mg caffeic acid equivalents (CAE) on 100 g DM.

Total flavonoids content (TFC) was determined using a method described by Issa-Issa et al. [21]. A 0.5 cm^3^ aliquot of sample was mixed with 0.1 cm^3^ of 10% (*w*/*v*) ethanolic solution of aluminum chloride, 0.1 cm^3^ of 1 M sodium acetate, and 4.3 cm^3^ of distilled water. After 30 min in the dark, the absorbance at 415 nm was spectrophotometrically measured. A calibration curve was built using quercetin (R^2^ = 0.997). Flavonoid content (expressed in mg quercetin equivalent (QE) per 100 g DM.

Total polyphenol content (TPC) was determined by spectrophotometric method using Folin–Ciocalteu reagent [22]. A total of 2 cm^3^ distilled water, 0.2 cm^3^ Folin–Ciocalteu reagent and 1 cm^3^ 20% Na_2_CO_3_ solution (m/m) were added to 0.1 cm^3^ of the extract. Absorbance was measured at wavelength, λ = 765 nm. Total polyphenol content was expressed in mg GAE/100 g DM (GAE—gallic acid equivalent).

All markers were made in three repetitions.

#### 2.2.6. Statistical Analysis

The obtained results were subjected to statistical analysis including two-way ANOVA. In order to determine the significance of differences between the average values, Duncan’s test was performed at a significance level *p* < 0.05. Additionally, between the parameters characterizing the properties of the nuts, the values of Pearson’s correlation coefficients were calculated, the significance of which was tested at the level of *p* < 0.01. Additionally, the principal component analysis (PCA) was performed. Statistical analysis was performed using Statistica 13.3 (StatSoft, Krakow, Poland).

## 3. Results and Discussion

### 3.1. Nutritional Value

Table 1 presents the parameters describing the nutritional value of the wheat breads enriched with hazelnuts and walnuts. The two-way analysis of variance confirmed the impact of the type of nuts, the level of enrichment and the interaction of both factors on the level of the average contents of total minerals, protein, fiber and fat (*p* < 0.001). It was determined that the enrichment of wheat breads with hazelnuts and walnuts increased the average level of mineral components (ash). The average total mineral content in wheat breads enriched with hazelnuts and walnuts was about 170% and 400% higher, respectively, compared to the control sample (0.32 mg/100 g DM). Thus, the enrichment of the bread with walnuts almost doubled the ash content. On the other hand, wheat bread enriched with walnuts was characterized by the highest ash content, of 9%. The increased level of total minerals in enriched breads is likely due to a lower proportion of flour in the dough recipe and thus an increased proportion of hazelnuts and walnuts. According to Venkatachalam and Sathe [23], both hazelnuts and walnuts, especially the seed coat, are very good sources of minerals. Therefore, the enrichment process had a very positive effect on the content of beneficial minerals. Nutritional value is also determined by protein. Similar to the mineral components, the process of bread enrichment with nuts resulted in an increase in protein content. The average content of this component in breads enriched with hazelnuts was 14.77 g/100 g DM and in breads enriched with walnuts was 15.24 g/100 g DM. Breads with a 9% nut content were characterized by the highest protein content, and the resulting breads did not differ statistically significantly in terms of this parameter. The enrichment process also had a significant statistical effect on the fiber content. The content of the described parameter in bread increased statistically significantly as the nut content in the recipe increased. The fiber content in the control sample was 1.49 g/100 g DM, whereas in breads enriched with hazelnuts it was statistically significantly higher, by 0.07 g/100 g DM on average, and in breads with walnuts, by 0.17 g/100 g DM. Dietary fiber is a very important food ingredient in terms of its health-promoting properties. Increasing the amount of fiber in food products, and as a result, its supply in the diet, has a positive effect on the functioning of the digestive system and promotes protection against cancer. Fat is the main component found in nuts. The process of adding nuts to enrich the bread also increased its fat content. The average fat content in breads enriched with hazelnuts was 1.53 g/100 g DM, which was about 5% higher than the control bread on average. In the case of breads enriched with walnuts, the increase in fat content compared to the control sample was above 8%. We can state that the process of bread enrichment with nuts increased the nutritional value of wheat bread. Compared to cereal seeds, nuts are characterized by a favorable chemical composition as they do not contain gluten proteins, have a higher fiber content and a very high fat content with a favorable chemical composition [23]. Furthermore, the fat from nuts is valuable because, according to Ros and Mataix [24], walnuts are the richest source of polyunsaturated fatty acids; they have the most favorable ratio of n-6 and n-3 fatty acids, which should be 4:1. Hazelnuts, on the other hand, contain the most monounsaturated fatty acids of all nuts. The literature on the subject provides data on the influence of bread enrichment with seeds, including oilseeds, on their nutritional value. Kaszuba et al. [6] claimed that the results of adding pumpkin, sunflower and flax seeds to triticale bread increased the contents of protein, fat and minerals. The quoted researchers calculated that the addition of pumpkin seeds resulted in an increase in protein and mineral content by 20% and 36%, respectively, and the addition of sunflower seeds resulted in a 4-fold increase in the fat content of bread compared to the control sample. In addition, other researchers [25] found an increase in the levels of protein, fat, minerals and dietary fiber in gluten-free rolls due to the addition of oil flax seeds.

### 3.2. Laboratory Bread Baking Parameters

Table 2 presents the parameters of the baked wheat breads enriched with hazelnuts and walnuts. The two-way statistical analysis showed a significant statistical effect by the type of nut, level of enrichment and the interaction of both factors on the value of dough yield, bread volume and specific volume. The enrichment of wheat bread with nuts resulted in a significant increase in dough yield. The average yield value was 165.74% for the dough with hazelnuts and 168.89% for the dough with walnuts. The dough yield with walnuts was about 5% higher than with the control sample. Moreover, the yield of bread increased due to nut enrichment. The value of this parameter for bread with hazelnuts and walnuts was 2.5% and 2% higher, respectively, compared to the control sample. Hazelnut enrichment was shown to increase the bread yields more than walnut enrichment. Bread yield is a feature that depends on many factors, including the quality of flour, the way the dough is handled and fermented, the technological additives used, the moisture content of the dough, the weight of the billets and the type of bread [26]. The parameter clearly describing the baking value of the flour, but also the influence of the technological additives used on this value, is the volume of bread. The volume of bread was found to decrease statistically significantly as a result of the enrichment process, with increases in the share of nuts of both types in the recipe. The control bread had a volume of 566.6 cm^3^. Breads enriched with hazelnuts had a smaller volume than the control, by about 107.4 cm^3^ on average. Breads enriched with walnuts had a volume of only about 427.15 cm^3^, or about 25% less than the control bread. A significant negative linear correlation was found between the bread volume and specific volume, dough yield, total polyphenol content (TPC), TFC, TPAC, protein and fat content (r = −0.85, r = −0.82, r = −0.85, r = −0.72, r = −0.73, r = −0.71, r = −0.81, *p* < 0.01). Gomez et al. [1] observed an increase in the volume of wheat bread enriched with walnut flour, but only by 10%. With bread enrichment of more than 10%, the quoted authors observed a significant decrease in the bread volume. Almoraie [27] also observed a reduction in bread volume as a result of replacing part of the flour with walnut flour. Similar observations were made by Kaszuba et al. [6], who enriched triticale bread with oilseed. The reduction in bread volume due to the enrichment with nuts may result from a reduced proportion of gluten proteins, as a result of replacing part of the flour with the ground hazelnuts and walnuts. Almoraie et al. [27] estimated that the supplementation of bread with oilseed flour, including walnut flour, at a level higher than 20% has a very negative effect on its weight and volume. Chochkov et al. [28] claim that the optimal addition of walnut flour to maintain the correct loaf volume is 5–10%. Such an addition also provides a good sensory profile and increased nutritional value. Increasing the share of gluten-free proteins, fiber and fat in the bread also has a negative impact on its volume. This triggers interactions between these components and the gluten proteins, resulting in a weakening of the gluten network responsible for the retention of fermentation gases produced by the yeasts [1]. Sluimer et al. [29] claim that a small addition of fat to the dough improves its resilience and as a result, makes it possible to reach the required volume after baking. High levels of fat addition reduce the dough elasticity, and there is a reduced availability of water for starch pasting and reduced yeast activity. This consequently reduces the achievable volume of the loaves. Moreover, according to Colaric et al. [30], the smaller volume of bread enriched with nuts, especially walnuts, may be due to their antimicrobiological properties. This weakens the yeast responsible for the fermentation process. This is consistent with the results obtained in this study. Dallman’s number, being an indicator of the overall evaluation of bread, was significantly higher in the case of the control bread and the breads enriched with nuts to the least extent (Table 2). The uneven porosity of the bread crumb enriched with hazelnuts (6%, 9%) and walnuts (9%) is probably due to large amounts of fat hindering yeast activity and gas bubble retention, and the reduced amount of gluten in the dough as a result of replacing wheat flour with ground nuts. As a consequence, the three-dimensional gluten network supporting the bubbles was weakened.

### 3.3. Bread Crumb Color

Table 3 presents the parameters for evaluating the crumb color of the wheat bread enriched to various extents with walnuts and hazelnuts. The two-way analysis of variance showed a significantly statistical effect of the type of nuts, the level of bread enrichment and the interaction of both these parameters on the color parameters. The value of parameter L* describing the brightness of the sample decreased statistically significantly as the share of either type of nut in the bread recipe increased. The average value of this parameter was 67.17 for the bread enriched with hazelnuts and 57.83 for the bread enriched with walnuts. Thus, the enrichment of the bread with walnuts decreased the value of the brightness parameter (L*) by about 14% compared to the bread enriched with hazelnuts. Other authors made similar observations. Anil et al. [31] also observed a decrease in L* for the bread crumbs supplemented with a roasted hazelnut coat. The value of this parameter decreased along with the increase in the share of nut coat in the bread. For the sensory assessment, Almoraie [27] noted a darker color of the wheat bread crumb supplemented with nut flour. This was explained by the coloring of the nuts. Kaszuba et al. [6] also reported a decrease in L* as a result of the enrichment of triticale bread with selected oilseeds (pumpkin, sunflower, flax seed). Another component of the color was parameter a*, whose value was 1.57 for the bread enriched with hazelnuts and 4.48 for the bread with walnuts. In the case of bread enriched with walnuts, the value was about three times larger than for bread enriched with hazelnuts. It is worth noting that a* had a statistically significant increase as the share of walnuts in the bread recipe increased, which means that the crumb became increasingly red. The control test was characterized by the highest value of parameter b*, and the process of bread enrichment with hazelnuts and walnuts resulted in a significant decrease in this value. The calculated color difference of all three components (ΔE) ranged between 0.75 and 6.39 for the bread with hazelnuts and between 6.00 and 15.86 for the bread with walnuts. According to Kowalczewski et al. [19], a ΔE of the crumb greater than 3 allows even an untrained observer to note differences in crumb color. A significant negative linear correlation was found in the study between L* and a*, ΔE, TPC, TFC, TPAC, as well as the protein and ash content (r = −0.87, r = −0.93, r = −0.77, r = −0.83, r = −0.76, r = −0.76, r = −0.73, *p* < 0.01, respectively). On the other hand, L* was significantly positively correlated with the values of b* and bread crumb gumminess (r = 0.90, r = 0.83, *p* < 0.01). The change in the color of the bread crumb due to the enrichment with nuts stems from the decreasing share of wheat flour in the recipe and from the color of the nuts. Nuts, and especially the coat of hazelnuts and walnuts, contain carotenoid pigments and flavonoids that shape their color. Amoraie [27] also observed the darkening of wheat bread when part of the flour was replaced with walnut flour. This was explained by referring to the color of the walnuts. This was also confirmed by Anil et al. [31], who also explained the darkening of the crumb color of bread supplemented with hazelnut coats by the dark, natural color of nuts and the process of hydration of the fiber present in the coat. The color of the bread crumb may also be related to the caramelization process during baking and the Millard reaction between the asparagine amino acid and reducing sugars.

### 3.4. Bread Texture Parameters

The mechanical properties of the bread crumb are qualitative characteristics that provide information about the texture of the product. Textural properties determine the durability of a product and its acceptability to consumers. One of the most frequently determined parameters when assessing the texture of bread is hardness. It depends on many factors, such as the type of additives used, the way the dough is handled and the specific weight of the bread crumb [32]. The two-way analysis of variance showed a significantly statistical effect of the type of nuts, the level of bread enrichment and the interaction of both these parameters with the values of the parameters of texture, such as hardness, gumminess and chewiness. The crumb of the control bread (9.23 N) was characterized by the lowest hardness, whereas the process of bread enrichment resulted in a statistically significant increase in the value of this parameter (Table 4). The average hardness of the crumb of the bread with hazelnuts was about 23% higher than that of bread with walnuts. The enrichment of bread with nuts resulted in an increase in the amount of fiber (Table 1), which also affects the rheological properties of the dough. This dough has a higher adsorption of water, which determines the greater hardness and crumbling of the loaf [33]. The quoted authors state that the addition of non-bread flours increases the hardness of bread crumb. According to Li et al. [34], soluble fiber fractions (especially arabinoxylans) can increase the stability of the dough, because the fibers combine in the form of chains with other macromolecules. As a result, it probably increases the hardness of the crumb. Similarly, Kaszuba et al. [6] noticed a significant increase in the crumb hardness of triticale bread due to the enrichment with ground pumpkin, sunflower and flax seeds. Lian et al. [35] found a 45% increase in the hardness of the gluten-free bread by adding chickpea flour and soy flour. The study did not show any significant influence of the statistical type of nuts, the level of bread enrichment and the interaction of both these parameters on the cohesiveness values. The bread crumb with a 9% walnut content was the most cohesive sample and control crumb was the least cohesive (Table 4). Only the type of nuts used to enrich the bread had a significant statistical effect on the elasticity of the bread crumb. The average elasticity of the bread crumb enriched with hazelnuts was 12.4 mm, slightly more than 2% higher than the average elasticity of bread with walnuts. The control crumb was characterized by the lowest gumminess, and bread crumb with a 3–9% hazelnut content by the highest. The case was similar with chewiness, where the crumb of the bread with a 6% hazelnut content achieved the highest value.

### 3.5. Bread Antioxidant Properties

Table 5 presents the values of parameters showing the antioxidant properties of wheat breads enriched to various extents with hazelnuts and walnuts. The two-way analysis of variance showed significant statistical effects related to the type of nut, the level of bread enrichment and the interactions of both these parameters on the values of antioxidant potential determined by the method using the ABTS* radical, total phenolic acids and flavonoids. Only the type of nut had a significant impact on the total polyphenol content. Other factors were insignificant. The value of the antioxidant potential determined by the ABTS* method increased statistically significantly as a result of nut enrichment and the increase in the amount of nuts in the product. The average value of antioxidant potential of breads enriched with walnuts was about 39% higher in comparison to bread containing hazelnuts. Wheat bread with a 9% walnut content was characterized by the highest antioxidant capacity (Table 5). The average content of phenolic acids in the control sample was 9.14 mg CAE/100 g DM, and the process of nut enrichment resulted in a significant increase in their content. Breads with walnuts (19.70 mg CAE/100 g DM) were characterized by the highest average content of phenolic acids. A small content of flavonoids was also found in the breads. Only 0.59 mg QE /100 g DM was present in the control sample, and as in the case of the previous parameters, the content of flavonoids increased statistically significantly as the nut content in the recipe increased, although it also depended on the type of nuts added. The average content of flavonoids in bread samples enriched with walnuts was about 2.5 times higher than their content in bread samples with hazelnuts. A similar trend was observed in the total polyphenol content. The enrichment of the bread with walnuts had a much greater effect on the level of total polyphenols than the addition of hazelnuts. The average content of total polyphenols in breads enriched with walnuts was 35.77 mg GAE/100 g DM and was 10.39 mg GAE/100 g DM higher than the average content of polyphenols in breads with hazelnuts (Table 5). Dry walnut seeds are characterized by a high antioxidant potential and total polyphenol content. By analyzing the content of polyphenols in the walnuts of 11 walnut varieties, Pycia et al. [36] finds that the average total polyphenol content (TPC) is 1.47 g GAE/100 g DM. Moreover, on analyzing the total polyphenols content in nuts of different botanical origin, Kornsteiner et al. [5] indicated that walnut seeds have a higher polyphenol content (1.62 g GAE/100 g DM) compared to hazelnut seeds (0.29 g GAE/100 g DM). This confirms the greater antioxidant potential of walnut-enriched bread. According to Sporin et al. [10], high temperature during baking has a very negative impact on the antioxidant potential and TPC. Nevertheless, Sivam et al. [37] maintain that the antioxidant and total TPC capacity of enriched bread is affected by the polyphenol content of flour, the polyphenols contained in the enriching ingredient, the Maillard reaction products with a strong antioxidant potential and the polyphenol–polysaccharide compositions produced during baking.

### 3.6. Practical Component Analysis

**Figure 1:**
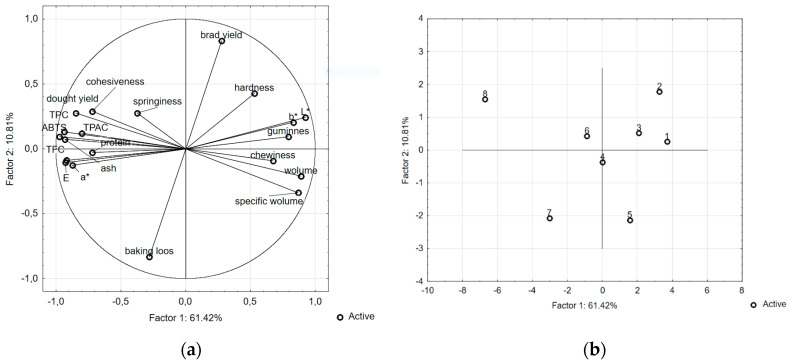
Practical component analysis: (**a**). the distribution of the analyzed parameters; (**b**). the distribution of the enriched bread samples (1–4—the breads enriched with hazelnuts at the levels of 1%, 3%, 6% and 9%, respectively; 5–8—the breads enriched with walnuts at the levels of 1%, 3%, 6% and 9%, respectively).

The analytical data obtained were statistically processed using cluster analysis and principal components analysis (PCA). The relationships between the input variables and the main components are shown in Figure 1a. This projection shows the distribution of the characteristics of particular analyzed parameters on the plane formed by two selected factors. PCA explains 83.73% of the total variability, with the first and second factors explaining 64.98% and 18.75% of the intergroup variances, respectively. The analysis of the presented distribution of the variables allows their division into two major groups. The first focuses on the parameters related to the texture of the bread crumb and its specific volume. The second group includes the parameters indicating mainly nutritional value, antioxidant properties and the color of the crumb of the bread. The arrangement of both parameter groups in close proximity to the circle indicates that a large part of the information contained in the input variable is carried by the main components. The arrangement of variables from the first and second category in relation to each other indicates their mutual negative correlation. This is confirmed by the Pearson correlation coefficients determined for each parameter.

Figure 1b shows the projection of bread samples enriched with walnuts and hazelnuts at different levels on factor planes. The analysis of the relationship between factor 1 and 2 shows significant similarities between the breads enriched with hazelnuts. The sample enriched with the highest dose of walnuts clearly differs from the others.

## 4. Conclusions

The enrichment of wheat bread with hazelnuts and walnuts has a significant statistical effect on its nutritional value, volume, crumb color, texture properties and antioxidant potential. The addition of nuts to bread resulted in an increase in the fiber and fat content. However, the breads enriched with hazelnuts and walnuts were characterized by a significantly smaller volume compared to the non-enriched bread. Decreasing the volume of enriched bread was also associated with an increase in the hardness, gumminess and chewability of the crumb. Bread supplementation with nuts also changed the color, as the crumb of enriched bread had a darker color than that of non-enriched bread. On the other hand, all the enriched breads were characterized by significantly stronger antioxidant properties and total polyphenol content, proportionally to the share of nuts in the recipe. Consequently, the process of wheat bread enrichment with walnuts and hazelnuts had a very positive effect on the nutritional value of the bread and its antioxidant properties described by, for example, the total polyphenol content. It follows that hazelnuts and walnuts are examples of excellent bio-regenerative raw materials which, when added to bread, can shape its health-promoting qualities. From the point of view of both optimal technological and antioxidant properties, the addition of hazelnuts or walnuts in the range of 3–6% is beneficial.

## Figures and Tables

**Table 1:** Nutritional value of gallic acid equivalent the wheat bread enriched with hazelnuts and walnuts.

Kind of Ingredient (%)	Ash Content (mg/100 g DM)	Protein Content (g/100 g DM)	Fiber Content (g/100 g DM)	Fat Content (g/100 g DM)
Control (0)	0.32 ^a^ ± 0.01	12.53 ^a^ ± 0.03	1.49 ^a^ ± 0.01	1.46 ^a^ ± 0.01
	**Hazelnuts**			
1	0.70 ^b^ ± 0.02	14.03 ^b^ ± 0.06	1.52 ^b^ ± 0.01	1.49 ^a^ ± 0.01
3	0.95 ^b^ ± 0.02	14.30 ^c^ ± 0.14	1.57 ^b^ ± 0.06	1.50 ^a^ ± 0.07
6	0.88 ^b^ ± 0.06	15.07 ^e^ ± 0.58	1.54 ^b^ ± 0.04	1.51 ^a^ ± 0.07
9	0.91 ^b^ ± 0.08	15.67 ^g^ ± 0.21	1.60 ^c^ ± 0.01	1.62 ^b^ ± 0.03
	**Walnuts**			
1	0.90 ^b^ ± 0.09	14.57 ^d^ ± 0.15	1.62 ^c^ ± 0.03	1.52 ^a^ ± 0.06
3	0.99 ^b^ ± 0.11	15.40 ^f^ ± 0.10	1.66 ^c^ ± 0.07	1.46 ^a^ ± 0.03
6	1.91 ^c^ ± 0.31	15.23 ^ef^ ± 0.14	1.64 ^c^ ± 0.02	1.51 ^a^ ± 0.01
9	2.50 ^d^ ± 0.13	15.86 ^g^ ± 0.60	1.72 ^d^ ± 0.06	1.82 ^c^ ± 0.09
	**Two-Factor ANOVA—*p***			
factor 1	<0.001	<0.001	<0.001	0.021
factor 2	<0.001	<0.001	<0.001	<0.001
factor 1 × factor 2	<0.001	<0.001	<0.001	<0.001

Mean values of the three replicates marked with the same letter in the column do not differ significantly at a significance level of *p* < 0.05. DM—dry mass. Factor 1—type of nuts, Factor 2—the enrichment level, Factor 1 × factor 2—interactions between the type of nuts and the enrichment level; ± standard deviation.

**Table 2:** Parameters of the laboratory baking of the wheat bread enriched with hazelnuts and walnuts.

Kind of Ingredient (%)	Dough Yield (%)	Total Baking Loss (%)	Bread Yield (%)	Loaf Volume (cm^3^)	Specific Volume (cm^3^/g)	Dallman Scale (°)
Control (0)	161.87 ^a^ ± 0.10	15.16 ^e^ ± 1.38	147.87 ^a^ ± 2.41	566.6 ^f^ ± 7.6	2.65 ^e^ ± 0.06	100
	**Hazelnuts**					
1	161.74 ^a^ ± 0.16	12.81 ^abc^ ± 0.61	151.98 ^bce^ ± 1.07	470.0 ^e^ ± 10.0	2.16 ^d^ ± 0.03	100
3	165.09 ^c^ ± 0.05	12.26 ^a^ ± 0.67	152.93 ^ce^ ± 1.17	456.7 ^d^ ± 7.6	2.07 ^c^ ± 0.06	90
6	169.47 ^e^ ± 0.10	13.51 ^bcd^ ± 0.53	150.75 ^bcd^ ± 0.92	438.6 ^bc^ ± 2.9	2.03 ^bc^ ± 0.07	80
9	166.67 ^d^ ± 0.00	13.65 ^bcd^ ± 0.24	150.52 ^bcd^ ± 0.46	431.8 ^b^ ± 2.8	1.99 ^b^ ± 0.01	80
	**Walnuts**					
1	163.50 ^b^ ± 0.01	13.84 ^cd^ ± 0.21	150.17 ^bc^ ± 0.41	446.7 ^bd^ ± 5.7	2.07 ^c^ ± 0.03	100
3	164.89 ^b^ ± 0.01	12.61 ^ab^ ± 0.47	152.32 ^ce^ ± 0.82	445.3 ^bd^ ± 0.2	2.04 ^bc^ ± 0.04	90
6	169.64 ^e^ ± 0.10	14.22 ^d^ ± 0.18	149.52 ^b^ ± 0.32	436.6 ^bc^ ± 2.9	2.03 ^bc^ ± 0.01	90
9	177.55 ^f^ ± 0.15	13.07 ^abc^ ± 1.04	151.52 ^bce^ ± 1.82	380.0 ^a^ ± 10.0	1.74 ^a^ ± 0.04	80
	**Two-Factor ANOVA—*p***					
factor 1	<0.001	0.119	<0.001	<0.001	<0.001	<0.001
factor 2	<0.001	<0.001	<0.001	<0.001	<0.001	<0.001
factor 1 × factor 2	<0.001	0.120	0.298	<0.001	<0.001	<0.001

Mean values of the three replicates marked with the same letter in the column do not differ significantly at a significance level of *p* < 0.05. Factor 1—type of nuts, Factor 2—the enrichment level, Factor 1 × factor 2—interactions between the type of nuts and the enrichment level; ± standard deviation.

**Table 3:** Color of the crumb of wheat bread enriched with hazelnuts and walnuts.

Kind of Ingredient (%)	L*	a*	b*	ΔE
Control (0)	67.86 ^f^ ± 0.58	1.70 ^b^ ± 0.06	18.82 ^f^ ± 0.16	-
	**Hazelnuts**			
1	68.12 ^g^ ± 0.60	1.75 ^b^ ± 0.03	18.26 ^f^ ± 0.14	0.75 ^a^ ± 0.21
3	71.35 ^h^ ± 0.26	1.50 ^a^ ± 0.03	17.85 ^e^ ± 0.13	3.72 ^c^ ± 0.08
6	67.49 ^f^ ± 0.22	1.55 ^a^ ± 0.01	16.96 ^d^ ± 0.11	1.89 ^b^ ± 0.15
9	61.73 ^d^ ± 0.09	1.51 ^a^ ± 0.01	16.90 ^d^ ± 0.10	6.39 ^d^ ± 0.06
	**Walnuts**			
1	63.87 ^e^ ± 0.45	1.82 ^c^ ± 0.04	14.36 ^c^ ± 0.33	6.00 ^d^ ± 0.51
3	57.44 ^c^ ± 0.31	4.25 ^d^ ± 0.03	12.02 ^a^ ± 0.15	12.66 ^e^ ± 0.32
6	55.89 ^b^ ± 0.30	6.21 ^f^ ± 0.02	13.00 ^b^ ± 0.23	14.13 ^f^ ± 0.28
9	54.13 ^a^ ± 0.22	5.67 ^e^ ± 0.06	11.88 ^a^ ± 0.03	15.86 ^g^ ± 0.17
	**Two-Factor ANOVA—*p***			
factor 1	<0.001	<0.001	<0.001	<0.001
factor 2	<0.001	<0.001	0.077	<0.001
factor 1 × factor 2	<0.001	<0.001	<0.001	<0.001

Mean values of the three replicates marked with the same letter in the column do not differ significantly at a significance level of *p* < 0.05. Factor 1—type of nuts, Factor 2—the enrichment level, Factor 1 × factor 2—interactions between the type of nuts and the enrichment level; ± standard deviation.

**Table 4:** Parameters of the texture of the wheat bread enriched with hazelnuts and walnuts.

Kind of Ingredient (%)	Hardness (N)	Cohesiveness (-)	Springiness (mm)	Gumminess (N)	Chewiness (N)
Control (0)	9.23 ^a^ ± 0.21	0.52 ^a^ ± 0.02	12.77 ^c^ ± 0.18	17.63 ^a^ ± 0.67	8.05 ^a^ ± 1.61
	**Hazelnuts**				
1	13.31 ^c^ ± 0.51	0.55 ^b^ ± 0.01	12.54 ^c^ ± 0.22	24.20 ^d^ ± 0.45	9.59 ^b^ ± 1.19
3	14.81 ^d^ ± 0.41	0.56 ^b^ ± 0.05	11.49 ^b^ ± 1.14	26.44 ^e^ ± 0.32	9.37 ^b^ ± 1.15
6	16.08 ^e^ ± 0.70	0.60 ^c^ ± 0.07	12.83 ^c^ ± 0.13	26.80 ^e^ ± 0.92	10.82 ^d^ ± 0.64
9	17.05 ^f^ ± 0.11	0.67 ± 0.05	12.77 ^c^ ± 0.17	26.49 ^e^ ± 0.71	8.24 ^a^ ± 1.72
	**Walnuts**				
1	12.28 ^b^ ± 0.50	0.56 ^b^ ± 0.01	10.45 ^a^ ± 0.40	21.92 ^c^ ± 0.25	10.26 ^c^ ± 1.51
3	12.67 ^b^ ± 2.51	0.61 ^c^ ± 0.09	12.58 ^c^ ± 0.50	20.77 ^b^ ± 0.68	9.28 ^b^ ± 1.41
6	12.46 ^b^ ± 1.49	0.58 ^bc^ ± 0.04	12.77 ^c^ ± 0.00	21.48 ^c^ ± 0.09	8.59 ^a^ ± 167
9	12.17 ^b^ ± 1.53	0.69 ^d^ ± 0.03	12.68 ^c^ ± 0.15	17.75 ^a^ ± 0.59	8.20 ^a^ ± 1.11
	**Two-Factor ANOVA—*p***				
factor 1	<0.001	0.641	<0.001	<0.001	<0.001
factor 2	<0.001	0.048	0.503	<0.001	<0.001
factor 1 × factor 2	<0.001	0.937	0.253	<0.001	<0.001

Mean values of the three replicates marked with the same letter in the column do not differ significantly at a significance level of *p* < 0.05. Factor 1—type of nuts, Factor 2—the enrichment level, Factor 1 × factor 2—interactions between the type of nuts and the enrichment level; ± standard deviation.

**Table 5:** Antioxidant properties in the wheat bread enriched with hazelnuts and walnuts.

Kind of Ingredient (%)	ABTS (µmol TE/100 g DM)	TPAC (mg CAE/100 g DM)	TFC (mg QE/100 g DM)	TPC (mg GAE/100 g DM)
Control (0)	30.84 ^a^ ± 0.63	9.14 ^a^ ± 0.74	0.59 ^b^ ± 0.01	8.31 ^a^ ± 0.78
	**Hazelnuts**			
1	37.81 ^b^ ± 1.54	9.30 ^a^ ± 1.45	0.61 ^a^ ± 0.08	26.09 ^b^ ± 2.46
3	41.86 ^c^ ± 1.55	12.84 ^bc^ ± 1.69	0.75 ^a^ ± 0.05	24.00 ^b^ ± 0.90
6	45.23 ^d^ ± 2.72	14.12 ^c^ ± 2.50	0.64 ^a^ ± 0.03	26.01 ^b^ ± 1.34
9	52.89 ^f^ ± 1.82	12.67 ^bc^ ± 1.14	0.66 ^a^ ± 0.08	25.31 ^b^ ± 0.45
	**Walnuts**			
1	41.54 ^c^ ± 1.05	10.43 ^ab^ ± 0.28	1.02 ^b^ ± 0.05	30.80 ^bc^ ± 3.60
3	49.04 ^e^ ± 0.56	14.92 ^c^ ± 0.56	1.48 ^c^ ± 0.03	34.94 ^c^ ± 2.11
6	68.99 ^g^ ± 1.37	23.76 ^d^ ± 0.96	3.19 ^d^ ± 0.05	37.92 ^d^ ± 1.57
9	87.88 ^h^ ± 1.96	29.70 ^e^ ± 1.95	3.49 ^e^ ± 0.18	39.45 ^d^ ± 0.88
	**Two-Factor ANOVA—*p***			
factor 1	<0.001	<0.001	<0.001	<0.001
factor 2	<0.001	<0.001	<0.001	0.073
factor 1 × factor 2	<0.001	<0.001	<0.001	0.046

Mean values of the three replicates marked with the same letter in the column do not differ significantly at a significance level of *p* < 0.05. ABTS*—(2, 2′-azinobis (3-ethylbenzothiazoline-6-sulfonic acid)); DM—dry mass; TE—Trolox Equivalent-α-tocopherol analogue; TPAC—total phenolic acids content; CAE—caffeic acid equivalents; TFC—total flavonoids content, QE—quercetin equivalent; TPC—total polyphenol content; GAE—gallic acid equivalent. Factor 1—type of nuts, Factor 2—the enrichment level, Factor 1 × factor 2—interactions between the type of nuts and the enrichment level; ± standard deviation.
